# Bio-ModelChecker: Using Bounded Constraint Satisfaction to Seamlessly Integrate Observed Behavior With Prior Knowledge of Biological Networks

**DOI:** 10.3389/fbioe.2019.00048

**Published:** 2019-03-26

**Authors:** Hooman Sedghamiz, Matthew Morris, Travis J. A Craddock, Darrell Whitley, Gordon Broderick

**Affiliations:** ^1^Center for Clinical Systems Biology, Rochester General Hospital, Rochester, NY, United States; ^2^Institute for Neuro Immune Medicine, Nova Southeastern University, Fort Lauderdale, FL, United States; ^3^Departments of Psychology and Neuroscience, Computer Science, and Clinical Immunology, Nova Southeastern University, Fort Lauderdale, FL, United States; ^4^School of Computer Science, Colorado State University, Fort Collins, CO, United States; ^5^Department of Biomedical Engineering, Rochester Institute of Technology, Rochester, NY, United States

**Keywords:** multi-valued discrete logic, constraint satisfaction, regulatory networks, multi-objective, data compliance, transition efficiency, path robustness

## Abstract

The *in silico* study and reverse engineering of regulatory networks has gained in recognition as an insightful tool for the qualitative study of biological mechanisms that underlie a broad range of complex illness. In the creation of reliable network models, the integration of prior mechanistic knowledge with experimentally observed behavior is hampered by the disparate nature and widespread sparsity of such measurements. The former challenges conventional regression-based parameter fitting while the latter leads to large sets of highly variable network models that are equally compliant with the data. In this paper, we propose a bounded Constraint Satisfaction (CS) based model checking framework for parameter set identification that readily accommodates partial records and the exponential complexity of this problem. We introduce specific criteria to describe the biological plausibility of competing multi-valued regulatory networks that satisfy all the constraints and formulate model identification as a multi-objective optimization problem. Optimization is directed at maximizing structural parsimony of the regulatory network by mitigating excessive control action selectivity while also favoring increased state transition efficiency and robustness of the network's dynamic response. The framework's scalability, computational time and validity is demonstrated on several well-established and well-studied biological networks.

## Introduction

With the rapid advances in broad-spectrum biological assays and corresponding algorithmic developments in the computational sciences, the *in silico* analysis of regulatory networks has become an increasingly valuable tool in creating new insight into the underpinnings of complex biological phenomena. While conventional continuous domain models offer high temporal and state resolution these come at the cost of much more strenuous requirements with regard to data quantity and quality. Discrete logical modeling of regulatory networks offers a simple framework for the qualitative representation of complex dynamic behavior exhibited by even relatively simple biological networks. Such formalisms can be classified into two general categories: binary and multi-valued. Of the many proposed discrete modeling formalisms (see de Jong, 2002; Saadatpour and Albert, 2016) for an overview), the Generalized Discrete Framework (GDF) introduced by Thomas and D'Ari (1990) is one of the richest and most flexible methods. In this formalism, every biological entity (cell type, transcript, etc.) may assume an expression level proportional to the number of control actions it exercises in the regulatory network. Biologically, this mimics the diverse selective actions supported through a broad range of varying molecular receptor affinity. Furthermore, the context-specific nature of biological network dynamics is captured in the model by a set of tunable logical operators (K parameters) that provide high flexibility in response to multiple competing stimuli, each with different relative biological activity i.e., signal strength. There are many challenges in reverse engineering regulatory networks from quantitative (e.g., time sampled measurements) and qualitative information (e.g., behavior of steady states), these include;

- *Sparse, irregularly collected and incompletely surveyed samples*: Often experimental measurements are non-uniformly sampled and some markers may be very difficult to survey. Cost and sample availability limitations also lead more often than not to small sample size.
- *Large and complex networks*: The size of the network model parameter search space increases exponentially with respect to the number of entities and interactions, many of which may be unknown.
- *Multiple competing models*: With model complexity typically greatly exceeding the number of data records, there usually exist many model parametrizations that equally satisfy the limited experimental data.

Many of the early efforts (Bernot et al., 2004; Batt et al., 2011; Klarner et al., 2012b; Monteiro and Chaouiya, 2012) at parameterization of regulatory networks relied on NuSMV (Cimatti et al., 2000) to address the combinatorial nature of this problem. NuSMV relies on Ordered Binary Decision Diagrams (OBDD) for model checking that is known to have unpredictable memory allocation (Kurshan, 2018). Therefore, as soon as the number of entities or nodes in the model grows, it becomes increasingly difficult to rely on OBDD-based approaches. Alternatively, Klarner et al. (2012a) proposed a model identification method for multi-valued regulatory graphs based on a colored model-checking (Barnat et al., 2012) of general Linear Temporal Logic (LTL) to handle the combinatorial complexity of the parameter space. Their approach consists of first identifying those parameter sets that satisfy a set of observed experimental data and second ranking them based on *Length Cost* and *Robustness*. However, since the parameter space of multivalued regulatory networks is super exponential their approach is likewise limited to models with a small number of entities or nodes (≈ <15 binary nodes). Streck and Siebert (2015) improved the efficiency of LTL-based model checking by proposing a more efficient encoding method for biological time series. Likewise, Corblin et al. (2012) employed Answer Set Programming (ASP) in order to infer dynamical properties in incomplete gene regulatory networks from incomplete expression data. But, it is not clear how scalable their proposed method is and whether different state transition update schemes such as synchronous and asynchronous are accommodated in their framework.

There are already various tools developed for logical analysis of biological networks, such as Caspo (Guziolowski et al., 2013), TREMPPI (Streck et al., 2016), GINsim (Chaouiya et al., 2003), and Bio Model Analyzer (BMA) (Benque et al., 2012). Caspo employs ASP in order to parameterize the regulatory networks. However, it is only applicable to Boolean models and only minimizes the Mean Squared Error (MSE) to experimental measurements. TREMPPI is able to parameterize multivalued models, but being based on LTL model checking it does not scale to larger networks (≈40 binary nodes or more) (Streck, 2015) and also does not incorporate the derivation of some features supporting the use of multi-valued models such as thresholds of action which are very hard to estimate a priori. GINsim also employs Thomas formalism in order to study the regulatory networks, but it is not able to learn the full trajectory dynamics directly from the experimental measurements. Similarly, BMA also applies model checking to multi-valued LTL networks (Claessen et al., 2013), making it accessible to non-programmers through a natural language and graphical interface (Ahmed et al., 2017), however its application is focused on recovery of end-point stable states in a synchronous updating environment. Furthermore, these tools are generally directed at supporting the manual entry of user-defined networks which limits the scale of the networks studied. In this work we attempt to build on the strengths of these various tools to produce a more integrated flexible environment that supports asynchronous updating and biological uncertainty, captures experimentally measured transition states and projects these onto larger networks where a user's prior knowledge is supplemented by the direct incorporation of a much broader automated text mining of the scientific literature conducted using Elsevier's (Amsterdam) MedScan natural language processing (NLP) engine (Novichkova et al., 2003).

In control theory the quality of a model parametrization is measured by its goodness-of-fit to data and structural parsimony. For instance, Akaike Information Criterion (AIC) combines the log-likelihood of adhering to the data and the model complexity (e.g., number of coefficients in a model) to assess the quality of a fit (Box et al., 1994). In logical regulatory models, especially as it might apply to the State Transition Graph (STG) generated by a model, goodness-of-fit might be understood as a combination of minimum number of transitions that is required to reproduce the time sampled measurement data and how robust that generated transition might be, while complexity can be interpreted by the number of interactions (e.g., edges) and thresholds of action (e.g., edge weights in multi-valued networks). This paper presents an extension of earlier conceptual work by our group (Sedghamiz et al., 2017) and the implementation of these formalisms into a standalone software tool capable of performing model checking and ranking regulatory networks: the Biological Model Checker (Bio-ModelChecker). First, a set of propositional equations inspired from Garg et al. (2008) are introduced for asynchronous and synchronous discrete GDF networks. After defining the logical equations for each specific time update scheme, the problem is formulated as a Constraint Satisfaction Problem (CSP). CSP offers a declarative and efficient way of describing combinatorial problems in terms of a set of constraints (Barták, 1999). This is particularly suitable for biological regulatory networks that need to satisfy a broad range of constraints. We construct bounded propositional formulas that allow us to efficiently check whether a regulatory model is able to reproduce a time sampled measurement. It is common to have several models reproduce a measurement equally well because the complexity of these networks more often than not greatly exceeds the number of available data records. Therefore, we extend the CSP problem into a multi-objective optimization. The method accepts an incomplete model from either automated natural language processing (NLP) or manual curation of literature as the first estimate of network structure and computes the complete set of parameterizations (topology and dynamics) of the model by maximizing several biologically inspired criteria namely: efficiency, robustness, and selectivity. We have implemented our proposed framework in FlatZinc (Nethercote et al., 2007; Becket, 2008) which is a standard CSP language readable by many state-of-the-art solvers. This gave us the ability to validate the proposed framework on several different solver technologies including Lazy Clause Generation (LCG) and Satisfiability Modulo Theories (SMT) (Yordanov et al., 2013; Giacobbe et al., 2017). Therefore, in this study, we employed three different solvers namely, Chuffed (Chu et al., 2014), Google Operations Research Tools (OR-Tools) (Perron, 2011) which employ LCG technology and OptiMathSat (Sebastiani and Trentin, 2015) that employs SMT. Our proposed method is validated on several biological regulatory networks detailed in Table 1.

**Table 1:** Benchmark problem definition.

**Network**	**|*V*|**	**|*E*|**	***F***	**|Ŵ|**	**|Û|**	**|$\hat{P}$|**	**|$\hat{K}$|**	***M***	
								**Synch**	**Asynch**
HPA	4	8	14	8	2	8	46,656	10	5
IRMA	6	9	16	0	0	0	≈604 x 10^6^	50	12
Dcell	114	129	3	0	0	0	95,268 × 10^135^	5	3
HPG	5	25	16	25	18	25	3^160^	3	8
Th	23	35	3	0	0	35	2^100^	2	2

Specification of the five benchmark problems to which an analysis was applied^*^, where |V| denotes the number of network nodes, |E| the number of regulatory edges, Fthe number of samples, |Ŵ| the number of activation thresholds supporting multi-level logic, |Û| the size of polarity specifier set, |$\hat{P}$| the size of the edge confidence set, and |$\hat{K}$| the size of the logical parameter space. M is the bounded horizon of reachable states along a trajectory.

^*^Stress hormone hypothalamic-pituitary-adrenal (HPA) axis (Sedghamiz et al., 2018); synthetic in vivo Reverse-engineering and Modeling Assessment (IRMA) network (Cantone et al., 2009); the dendritic cell cycle from Garg et al. (2008); the female sex hormone hypothalamic-pituitary-gonadal axis (HPG) (Bennett et al., 2013; Sedghamiz et al., 2017); T-helper cell differentiation (Garg et al., 2008). Note that the bound for model checking is different under synchronous and asynchronous update schemes.

This work is novel in several respects. Importantly it introduces a complete framework for the identification of biologically relevant parameters in a discrete logic regulatory network (i.e., a parsimonious topology, contextual decision weights, polarity of interactions, and even the threshold of actions among entities). Since we use a generalized Thomas framework it easily supports Boolean logic, multivalued logic, and the combination of both. The framework formulates the parameter identification problem as a bounded constraint satisfaction problem, enabling one to parametrize larger models by reducing the corresponding bound, something which remains daunting (NP-hard) in a conventional OBDD-based framework (Bollig and Wegener, 1996). It then ranks models satisfying these constraints based on their goodness-of-fit and complexity which in this discrete logic framework are denoted as path-length, robustness, number of interactions, and their threshold of action. Beyond the immediate task of parameter identification, our framework can also be used for model reduction, identifying the minimum number of interactions required to reproduce a desired behavior. Moreover, the whole framework is implemented in a unified standard constraint programming syntax which enables it to benefit from the latest state-of-the-art solvers which are well-supported and frequently updated.

## A Regulatory Network Model

In the following sections, first, we briefly review GDF and rigorously formulate the parametrization problem. Then, we introduce the multi-valued logical equations for synchronous and asynchronous update schemes. Next, we introduce the concepts of Efficiency, the Length Cost, Robustness, and Selectivity. A biological regulatory graph *G* = (*V,E,W,U,P*′) is a signed, weighted, and directed graph. Where:

- *V* denotes the entities (e.g., transcripts, proteins, cells, etc…) in the network,
- ρ_*i*_:*V* → ℕ_1_ is the maximum expression level that node *v*_*i*_ may assume over its domain *D*_*i*_. In an unconstrained network node, ρ_*i*_ is initially set by default to the number of actions component *v*_*i*_ exerts on the network (e.g., out-degrees).
- ℕ_1_ denotes the complete set of natural numbers excluding 0
- *E* ⊆ *V* × *V*, is the set of interactions (edges) where:
- *w*_*ij*_ ∈ {1, …, ρ_*j*_} is the *interaction threshold* above which the regulation from node *v*_*j*_ to *v*_*i*_ is active,
- *u*_*ij*_ ∈ {−1, 1} is a polarity associated with such an interaction where an activating (inhibiting) effect is expressed by *u*_*ij*_ = 1 (*u*_*ij*_ = −1),
- *p*_*ij*_ ∈ {0, 1}, is a bit associated with edge *w*_*ij*_ indicating that the existence of this edge is necessary for the network. This vector of bits ′*P* is intended to incorporate prior knowledge in the parameterization process.

### State Transition Function and Graph

Each component *v*_*i*_ ∈ *V* under the GDF is described by a set of logical parameters, *K*_*i*_:*y*_*i*_ → [0, ρ_*i*_] governing its response behavior toward the target state *y*_*i*_ under combinatorial incoming actions from other components in the network. Intuitively, it might be thought of synthesis to decay kinetics of entities influencing *v*_*i*_ (Thomas et al., 1995). The state transition function (image) explains the temporal evolution of the regulatory network using the *K*_*i*_ and is defined as Chaouiya et al. (2003), Devloo et al. (2003);

(1) $$\begin{array}{c} y_{i}=K_{i}\left(I_{a}\right)\;where\\ I_{a}:=\left\{i\in V|(i,j)\in E\wedge S^{u_{ij}}\left(x_{j},\;w_{ij}\right)\right\} \end{array}$$

**Figure 1:**
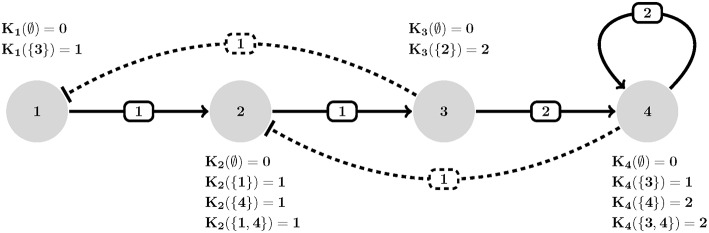
An example of HPA axis described in GDF. Each interaction (edge *e*_*ij*_) is assigned a threshold (*w*_*ij*_), polarity [*u*_*ij*_ where solid (resp. dashed) stands for positive (resp. negative)] and each node is allowed to assume an expression level equal to its number of actions (outdegrees). Transition dynamics of each node is represented by a set of *K*_*i*_(*I*) values. *K*_*i*_(∅) defines the basal value of entities (no activation is present). For instance, *K*_2_({1, 4}) defines how entity *v*_2_ behaves when both its activator *v*_1_ and inhibitor *v*_4_ are present simultaneously (which is an increase in its expression since *K*_2_({1, 4}) = 1). Adapted from Figure 1A, Sedghamiz et al. (2019).

$S^{u_{ij}}$is a threshold function that determines whether the expression level *x*_*j*_ of node *v*_*j*_ is sufficient to exercise a control action response i.e., activate (or inhibit) a regulatory target *x*_*i*_. Therefore, the set of all active interactions on a node are in fact each denoted by a unique *K*_*i*_(*I*_*a*_) logical value that collectively define the image of that node (see Figure 1). Given an instantiation of all topological (e.g., *W, V, U*) and dynamical (e.g., *K*) parameters, one can study the temporal evolution of regulatory graph G by iteratively computing the transition function in Equation (1). This would result in a State Transition Graph (STG) S that contains ∏_*i*∈*V*_(1+ρ_*i*_) states. Note that the edges in S (i.e., number of edges and reachability of states) directly depends on the update scheme employed during the simulation. The most popular update schemes reported in literature are synchronous and asynchronous (Kauffman, 1969; Thomas and D'Ari, 1990; Albert and Robeva, 2015).

## Parameter Identification Problem

The parameter identification problem consists in identifying from all parameter sets available to the model $\overset{G}{\sum }$, the sub-family of parameter sets ${\hat{\sum }}^{G}$ (where ${\hat{\sum }}^{G}$ ⊆ ∑^*G*^) that would enable a hypothetically incomplete regulatory network *G* to generate a certain behavior (e.g., produce a given attractor and/or a sequence of partial experimental measurements that might have to optionally satisfy other additional constraints).

### Parameter Space

We define the combinatorial parameter space of size $\left|\overset{G}{\sum }\right|$ associated with regulatory graph G as the product of all unknown model parameter subspaces for the following;

- *Logical values*:
  $$\left|K\right|=\prod_{i\in V}\left\{{\left(\rho_{i}+1\right)}^{2^{\left|q(i)\right|}}\right\}$$
  Where |*q*(*i*)| is the in-degree of component *v*_*i*_ [i.e., *q(i)* is the set of regulators of *v*_*i*_] and where there are 2^|*q*(*i*)|^ parameters required to capture all possible combinatorial effects on component *v*_*i*_ with each of those parameters assuming a value in [0, ρ_*i*_ ].
- *Threshold of action*:
  $$\left|W\right|=\prod_{i\in V}\prod_{j\in \left(q(i)\cap \hat{w}\right)}\rho_{j}$$
  Where *w*_*ij*_ → [1, ρ_*j*_] and where Ŵ ⊆ *W* denotes the set of interactions for which the threshold of action is not known *a priori*.
- *Polarity*:
  $$\left|U\right|=\prod_{i\in V}\prod_{j\in (q(i)\cap \hat{U})}2$$
  Where, *u*_*ij*_ ∈ {0, 1} and where Û ⊆ *U* denotes the set of interactions for which polarity is not known *a priori*. Note that in a multi-valued formalism, the polarity of an edge constrains the logical values involved in that interaction. For instance, a positive (resp. negative) edge from *v*_*j*_ to *v*_*i*_ requires that there exists at least a logical value that increases (respectively decrease) the expression level of *v*_*i*_ once *v*_*j*_ is active. Formally:
  $$S^{u_{ij}}\left(x_{j},\;w_{ij}\right)↔\exists R\subseteq q\left(i\right)\;:\;K_{i}(R)<K_{i}(R\cup j)$$
  Where *R* is a subset of *q(i)* the set of all regulators of node *v*_*i*_. Intuitively, this criterion requires that the regulators of node *v*_*i*_ should at least be weak activators or inhibitors. However, it is possible to apply a stricter constraint that requires the regulator of node *v*_*i*_ to be strong modulators (e.g., $S^{u_{ij}}\left(x_{j},w_{ij}\right)↔\forall R\subseteq q\left(i\right)\;:\;K_{i}\left(R\right)<K_{i}\left(R⋃j\right)$). In this study, we only require that the regulator be at least weak regulator but have the possibility to strengthen this constraint (see Klarner et al., 2012b for a more detailed list of available constraints).
- *Confidence of interaction*:
  $$\left|P\right|=\prod_{i\in V}\prod_{j\in \left(q(i)\cap \hat{P}\right)}2$$
  Where *p*_*ij*_ ∈ {0, 1} and where *P* is a bit vector with a cardinality equal to the total number of interactions in the network (*E*) and where a true bit in this vector implies that the existence of an interaction is necessary in order to satisfy a compliant parameterization. $\hat{P}\subseteq P$ denotes the subset of interactions that are poorly supported or absent altogether from prior knowledge and for which a high confidence level could not be assigned by the user. The vector *P* is useful for finding the most parsimonious model (e.g., by minimizing its cardinality) while incorporating established prior knowledge and exploring the possible role of new posited interactions.

Consequently, the parameter space of G is super-exponential:

$$\left|\sum^{G}\right|=\left|S\right|\times \left|K\right|\times \left|W\right|\times \left|U\right|\times \left|P\right|$$

Where |*S*| is the size of the state transition graph, |*K*| the size of the logical parameter space, |*W*|, the size of the threshold of action parameter space, |*U*| the size of the polarity of action space and |*P*| the size of the confidence of action space.

### Logical Equations

In the process of regulatory graph generation, sometimes it is not possible to confidently determine the polarity of the regulatory action (Û ⊆ *U*) and the thresholds of action (Ŵ ⊆ *W*). Furthermore, experiments are often sparsely sampled and incompletely surveyed (only partially observed). Therefore, our proposed method combines the partial information from the incomplete model and measurements in order to constrain the parameter sets to satisfy both as well as infer the unknown model parameters (*K*, Û, Ŵ) and the expected values of missing experimental measurements. In this section, we formally introduce equations specific to the logical time update scheme (synchronous and asynchronous).

#### Propositional Formula for State Transition Update Schemes

In order to study the temporal evolution of a biological system, we first need to formally derive the logical equations under each updating schedule. In this study, we derive such logical equations for two well-known synchronous and asynchronous update schedules. Let $x^{t}=\{x_{1}^{t},x_{2}^{t},\ldots ,x_{n}^{t}\}\;$ be a vector representing the state of a regulatory network, *TR*_*MxN*_ be a trajectory consisting *M* transitions **x**^**t**^is the state of the network at time *t* and *N* entities. The logical equation governing such a trajectory depends on the choice of update scheme is may be defined for the classical synchronous and asynchronous update schemes as follows:

- Under synchronous update all state variable nodes may update their current state simultaneously which can be denoted as the conjunction of transitions $T_{i}^{Synch}$across all elements *v*_*i*_:
  (2) $$TR_{1\ldots M}^{Synch}=\wedge_{t=1}^{M-1}\wedge_{i=1}^{N}T_{i}^{Synch}(x^{t},x^{t+1})$$
- Under asynchronous update only one state variable node at a time is permitted to update from its current state which can be denoted as the disjunction of transitions $T_{i}^{Asynch}$ across all elements *v*_*i*_:
  (3) $$TR_{1\ldots M}^{Asynch}=\;\wedge_{t=1}^{M-1}\vee_{i=1}^{N}T_{i}^{Asynch}\left(x^{t},x^{t+1}\right)$$

Where $T_{i}^{Synch}$ and $T_{i}^{Asynch}$represent the transition for node *v*_*i*_ given its image vector ***Y*** and current state of the network **x**^**t**^ under synchronous and asynchronous update, respectively. Additional details regarding the derivation of these propositional formulas may be found in the Supplementary Material.

#### Propositional Formula for Bounded Model Checking of Time Series

In the previous section, we stated that the output of a regulatory model might be logically formulated as the conjunction of several states. A time series might be denoted as a matrix *L*^*FxN*^ with *F* time samples and *N* entities. We say a model generates time series *L* where there exists at least a path in its STG which passes through all the samples in *L* sequentially (e.g., in order from time sample *t*_1_ to *t*_*F*_). This problem has been traditionally addressed with OBDD based symbolic model checking (Cimatti et al., 2000) where reachability analysis is performed on sets of states rather than individual states. However, the computational requirements and memory allocation associated with the identification of optimal (minimal node) OBDD does not scale well with increasing problem size even using the most efficient ordering techniques (Bollig and Wegener, 1996; Singh and Mohan, 2008). As a result, Bounded Model Checking techniques (Clarke et al., 2001) were introduced that instead of checking the reachability properties on sets of states deal with a user-defined bounded number of states that mitigate the state space explosion. These methods construct a propositional formula for which satisfiability is checked using SAT solvers. Given:

- A transition characteristic function defined under the synchronous (Equation 2) or asynchronous update scheme (Equation 3).
- A user supplied upper bound *M* on the number of transitions and
- A matrix of time series measurements L^*FxN*^ that might contain uncertain or unmeasured entities denoted by ⊥.

The *unrolled* propositional formula for a pair of samples in *L* (e.g., consecutive rows *l*_*f*_ and *l*_*f*+1_) is defined as follows:

- For the synchronous update scheme:
  (4) $$\begin{array}{l} {〚SS〛}_{(l_{f},l_{f+1})M}:\;=\;\left(\wedge_{i\in B_{f,j}}l_{f,i}↔x_{i}^{1}\right)\\ \;\wedge \left(\wedge_{t=1}^{M-1}\wedge_{i=1}^{N}T_{i}^{Synch}\left(x^{t},x^{t+1}\right)\right)\\ \;\wedge \left(\vee_{t=2}^{M}\wedge_{i\in B_{f,j}}l_{f+1,i}↔x_{i}^{t}\right) \end{array}$$

where,

$$B_{f,j}:=\left\{j\in \left[1,n\right]|l_{f,j}\neq ⊥\right\}$$

The first term of Equation (4) sets the initial state of the transition (x^1^) equal to the first pair of the sampled measurement (*l*_*f*_) for those entities which are certain (¬⊥). The second term computes the set of all reachable states within a bound *M*. Finally, the last term states that at least one of the transitions within the bound (*t* : = [2,M]) should be equal to the second pair of the measurement (*l*_*f*+1_).

- For the asynchronous update scheme similarly:
  (5) $$\begin{array}{l} {〚SS〛}_{(l_{f},l_{f+1})M}:=\left(\wedge_{i\in B_{f,j}}l_{f,i}↔x_{i}^{1}\right)\wedge (\wedge_{t=1}^{M-1}\vee_{i=1}^{N}T_{i}^{Asynch}\\ \;\left(x^{t},x^{t+1}\right))\wedge \left(\vee_{t=2}^{M}\wedge_{i\in B_{f,j}}l_{f+1,i}↔x_{i}^{t}\right) \end{array}$$

Note that Equations (4) and (5) apply to pairs of measurements only. For a measurement matrix with more than *F* > 2 samples, we construct conjunction of Equations (4) and (5) consecutively. Therefore, for a measurement matrix *L*^*FxN*^;

(6) $$\wedge_{f=1}^{F-1}SS_{\left(l_{f},l_{f+1}\right)M}$$

Intuitively, for each pair in *L* a set of all reachable sets within a bound *M* is computed and the whole matrix is reproducible when all sub-clauses in Equation (6) are satisfiable.

**Example**: Assume a measurement matrix *L* with 3 time-course samples of the simple generic example depicted in Figure 1 which contains three uncertain measurements:

$$L=\left[\begin{array}{cc} ⊥ & 0\\ 1 & ⊥\\ 1 & ⊥ \end{array}\;\begin{array}{cc} 1 & 0\\ 2 & 1\\ 1 & 2 \end{array}\right]$$

The corresponding unfolded propositional formula for this matrix's row 1 and 2 (e.g., time samples 1 and 2) with a checking bound of *M* = 3 under the synchronous update scheme is defined as;

$$\begin{array}{l} {〚SS〛}_{{\left(l_{1},l_{2}\right)}_{3}\;:=\;}\underset{i\in B_{f,j}}{\wedge }l_{f,i}↔x_{i}^{1}\;:\left(\neg x_{2}^{1}\wedge x_{3}^{1}\wedge \neg x_{4}^{1}\right)\;\wedge \\ \;T_{1}^{Synch}\left(x^{1},x^{2}\right)\;:(\left(x_{2}^{2}↔\left(x_{2}^{1}+SC_{2}^{1}\right)\right)\wedge \left(x_{3}^{2}↔\left(x_{3}^{1}+SC_{3}^{1}\right)\right)\\ \wedge \left(x_{2}^{4}↔\left(x_{4}^{1}+SC_{4}^{1}\right)\right))\wedge \\ T_{2}^{Synch}\left(x^{2},x^{3}\right)\;:(\left(x_{1}^{3}↔\left(x_{1}^{2}+SC_{1}^{2}\right)\right)\wedge \left(x_{3}^{3}↔\left(x_{3}^{2}+SC_{3}^{2}\right)\right)\\ \wedge \left(x_{4}^{3}↔\left(x_{4}^{2}+SC_{4}^{2}\right)\right)\;)\wedge \\ \overset{M}{\underset{t=2}{\vee }}\underset{i\in B_{f,j}}{\wedge }l_{f+1,i}↔x_{i}^{t}\;:\;(\left(x_{1}^{2}\wedge \left(x_{3}^{2}↔2\right)\wedge x_{4}^{2}\right)\\ \vee \left(x_{1}^{3}\wedge \left(x_{3}^{3}↔2\right)\wedge x_{4}^{3}\right)) \end{array}$$

The propositional formula for row 2 and 3 of L (e.g., *SS*_(_*l*_2_, *l*_3_)3__) is computed similarly. Taking the conjunction of these two propositional formulas as shown in Equation (6) would result in bounded checking of the whole matrix. Note that the existence of node and cyclic attractors might also be easily checked with a similar propositional formula where the initial and end state are identical. This is true for node attractors and transient cycles under the asynchronous update scheme, and node attractors as well as cyclic attractors under the synchronous update scheme (Dubrova and Teslenko, 2011).

### Constraint Satisfaction Problem

CSP is a declarative paradigm in which the problems are described in terms of their constraints (Jaffar and Maher, 1994; Barták, 1999; Tack, 2009). A constraint satisfaction problem is stated as:

- A set of *V* = {*v*_1_*, …, v*_*n*_} variables; where each variable *v*_*i*_ has a domain *D*_*i*_.
- A set of constraints that restrict the bound of each variable.

A solution to CSP is the assignment of each variable to a domain that satisfies all of the constraints. CSP has been previously used successfully in identification of attractors in large regulatory networks (Devloo et al., 2003). In this work, we have formulated the model identification task as a CSP and implemented it in a standard CSP language known as *FlatZinc* (Nethercote et al., 2007) that is readable by many state-of-the-art solvers. An extension of the basic CSP might be solved as an optimization problem where for each satisfying solution the value of an objective(s) is improved (e.g., branch-and-bound; Clausen, 1999).

### Optimization Objective Functions

In control and regression theory, there exists measures such as Akaike Information Criterion (AIC) that combine goodness-of-fit (e.g., log-likelihood) and complexity (e.g., number of coefficients) of the model to score the quality of a fit. Inspired from the same ideas, in this section, we introduce several biologically relevant objective functions employed in our parameterization to rank the constraint satisfying models (Sedghamiz et al., 2017). We denote our final multi-objective function as a vector **Z** which consists of three objectives in the case of the asynchronous update scheme and two under synchronous update scheme as detailed below.

#### Structural Efficiency

Our first assumption is that biology tends to be energy efficient and parsimonious in structure. In multivalued regulatory graphs, this is reflected as a minimal number of interactions (|*E*|) and aggregate threshold of actions (*W*). A regulatory network is energy efficient and parsimonious if it is able to generate a response behavior with as few control actions as possible. This is defined as:

(7) $$z_{1}:=\{\begin{array}{l} minimize\;\sum_{i=1}^{N}\sum_{j\in q(i)}p_{ij}w_{ij},\\ Subject\;to\;w_{ij}\in \left[1,p_{j}\right]. \end{array}$$

Where *P* vector is a binary mask (see A Regulatory Network Model). *p*_*ij*_ is true *iff* the existence of edge *p*_*ij*_ is necessary in the graph *G* in order to reproduce a behavior.

#### Path Length Cost

A sequence of time sampled measurements might be assumed to represent a trajectory with length *F*. The shortest possible walk from the first sample *t* to *t* + 1 is denoted by *F*_*m*_*t, t*+1__. Then, the length cost is defined as the smallest number of transitions (shortest walk) required by a parameterization to reproduce the time-sampled trajectory in question. For instance, if a measurement only consists of two samples *t*_1_ and *t*_2_, then we find a smallest value of *F*_*m*_1,2__ for which Equation (6) is satisfiable [specifically see the last clauses in Equations (4) and (5)]. The length cost for a measurement with *F* samples is defined as:

(8) $$z_{2}:=\;minimize\;\sum_{t=1}^{F-1}\left(F_{m_{t,t+1}}-2\right)$$

**Figure 2:**
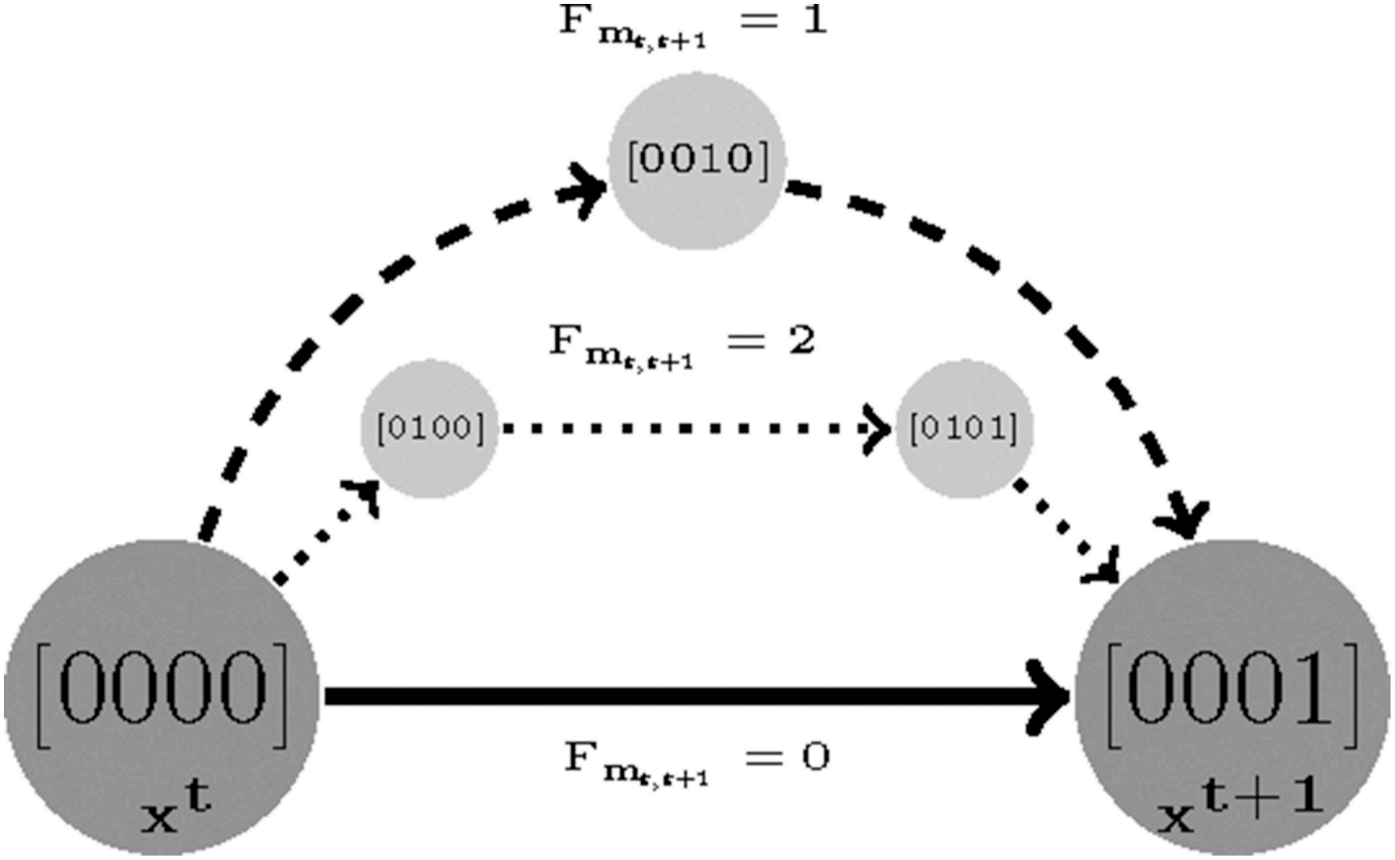
An example visualizing the length cost (denoted by *F*_*m*_*t, t*+1__) concept. Assuming (x^t^ and x^t+1^) are two sampled experimental measurements. There are three sets of parameters that make x^t+1^ reachable from x^t^: a direct transition with *F*_*m*_*t, t*+1__ = 0 and two indirect transitions with *F*_*m*_*t, t*+1__ = 2 and *F*_*m*_*t, t*+1__ = 1, respectively.

Where *F, F*_*m*_*t, t* + 1__ ∈ {1, …, *M*}, *M* ≤ *D*_*L*_ are the number of sampled measurements, the *minimum length cost* for samples *t* and *t* + 1, and reachability bound, respectively (*D*_*L*_ is the diameter of State Transition Graph (STG). Therefore, the minimum possible value for z_2_ is 0 (e.g., the constant 2 in Equation (8) is used to remove the initial and end state as offsets) and it grows as the length cost increases (see Figure 2).

#### Path Robustness

The asynchronous update scheme employed in this study considers the stochasticity in system behavior that results from uncertainty in the relative kinetics of the nodes in the network. As Klarner et al. (2012a) pointed out, variability in the time delays separating the sequential activation of different nodes manifests as perturbations to the system dynamics overall. A model is considered robust if it is insensitive to such changes. This can be quantified as the number of competing trajectories between time sample *t* to *t*+1.We prioritize the parameter sets that generate fewer branches between two consecutive measured states since as the number of paths increase the chance of deviating from the destination grows as well. We count the total number of variables that tend to change (*R*_*t, t*+1_) from state *t* to *t* + 1. Formally, we find a robust parameterization with respect to a set of measurements with length *F* by:

(9) $$z_{3}:=\{\begin{array}{l} minimize\sum_{t=1}^{F}R_{t,\;t+1}\;\\ where\;R_{t,\;t+1}=\sum_{i=1}^{N}\left(y_{i}^{t}⊕x_{i}^{t}\right)\\ subject\;to\;R_{t,\;t+1}\in \left[0,N\right] \end{array}$$

Where ⊕ is multivalued XOR operator (e.g., 2 ⊕ 1 = true; 3 ⊕ 3 = false).

**Figure 3:**
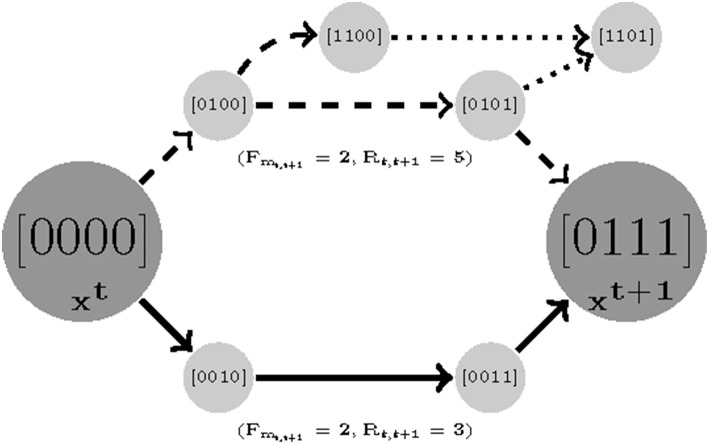
An illustration of robustness. Here there are two parameterizations that generate paths with equal length costs (solid and dashed). In order to compute the robustness, we only count the number of branches for the shortest trajectories. It is clear that while the dashed trajectory is still reachable to sample 2 with a similar cost length, it has a chance of deviating and missing its destination in transitions [0100] and [0101] (dotted branches).

The Robustness idea is illustrated in Figure 3. Note that it is a function of length cost. We first find a minimum *F*_*m*_*t, t*+1__ for which two samples are reachable and then for that *F*_*m*_*t, t*+1__ minimize the robustness cost. Note that the minimum value for *R*_*t, t*+1_ is 0, where 0 means that the state is steady (e.g., no variable tends to change). For an example see Figure 3, there are two different paths with equal length costs (solid and dashed curves) generated by two parameterizations (instantiation of *K*_*i*_(*I*), *W*, and *U*). The solid-line trajectory offers the possibility of an alternate destination and is hence has a higher robustness cost.

The multi-objective vector **Z** might be solved by linearly combining the objectives (e.g., $\overset{3}{\underset{i=1}{\sum }}\delta_{i}z_{i}$ where δ_*i*_ is a penalizing integer weight) or in a Pareto front manner which is supported by some of the solvers (e.g., OptiMathSat; Sebastiani and Trentin, 2015). In this study, we weighted all the objectives equally for the linear mixture.

## Benchmarks and Applications

As mentioned in the Introduction, the concepts reported here have been integrated into a standalone software tool, the BioMC (https://github.com/hooman650/BioModelChecker), that accepts the input problem definitions as a JSON file. The regulatory network structure is described by an adjacency matrix. This matrix has 5 rows, where the first and second row indicate the target and source node indices, respectively. The third and fourth rows indicate threshold of action and the polarity (e.g., activator/inhibitor), respectively. Finally, the last row is a binary flag that indicates whether the interaction should be necessarily preserved during the parameterization. This adjacency matrix may be defined directly by the user or may be generated automatically by deploying a text-mining tool to survey the scientific literature, or both. Currently our group uses the MedScan natural language processing (NLP) engine (Novichkova et al., 2003) that supports the Pathway Studio database (Elsevier, Amsterdam) to extract regulatory interactions reported in the literature. The output of the pathway studio is an adjacency list that indicates the target and source entities and their nature of interaction (e.g., activator/inhibitor). BioMC directly imports this list and completes the annotation by computing the regulatory polarity and the expected confidence metric for each edge (the last row of the adjacency list). The BioMC analytical framework is implemented for Windows 64bit and incorporates three solvers. In our implementation, we employ variable ordering by instructing the solvers to start with those variables having the most narrow range of discrete values. In this work, we also compare the results obtained from solving the objective vector **Z** by linearly combining its components with the Pareto approach proposed in Sebastiani and Trentin (2015). We demonstrate the capabilities of this framework by applying the latter to the 5 benchmark problems described in Table 1. Specifically, we show how the framework can be used to effectively integrate sparsely collected and partially observed samples with prior knowledge of the network structure in order to validate and rationalize the latter and support the mechanistically informed simulation of the system's dynamic behavior at various scales of biology and levels of structural complexity (i.e., connection density). All the sequential solvers were tested on an Intel core i7 machine.

### Model Validation: The Hypothalamic-Pituitary-Adrenal (HPA) Axis

The Hypothalamic-Pituitary-Adrenal (HPA) axis represented in Figure 1 is the most central regulator of immune and endocrine response to stress and has aptly been called the “fight or flight” axis. Due to its important regulatory role, it is no surprise that the HPA axis has been associated with a number of complex chronic diseases (Silverman and Sternberg, 2012). Perceived stress triggers a cascade of hormone release starting with corticotrophin-releasing hormone (CRH; node 1) and adrenocorticotropic hormone (ACTH; node 2) by the hypothalamus and pituitary in the mid-brain and leading to the release of the broad-acting immune regulator cortisol (node 3) from the adrenal glands and up-regulates receptor expression R (node 4). Circulating cortisol then regulates in a negative feedback to the mid-brain where it slows additional CRH (node 1) release (see also, Figure S2.1). Here we provided 7 time points simulated for two of the 4 state variables and asked the model checker to find all missing parameters (interaction polarities, threshold of actions, and dynamics) defining the 6 regulatory interactions. In addition, we introduced two spurious interactions to the model in order to see whether the model checker would be able to flag these (all the bits in confidence vector P corresponding to all interactions were set as unknown). In the synchronous case, all the three solvers agreed on a single solution that accurately aligns with the reference HPA axis model presented in our earlier work (Sedghamiz et al., 2018, also see Figure 1). Interestingly, this solution also correctly identified the 2 spurious interactions as redundant (e.g., interactions from the glucocorticoid receptor R (node 4) to corticotropin-releasing hormone CRH (node 1), and CRH (node 1) to cortisol (Cort; node 3), respectively). The time required to converge to a solution was very similar for *OR-tools* and *Chuffed*.

### Model Reduction: The T-Helper Cell Differentiation Network

**Figure 4:**
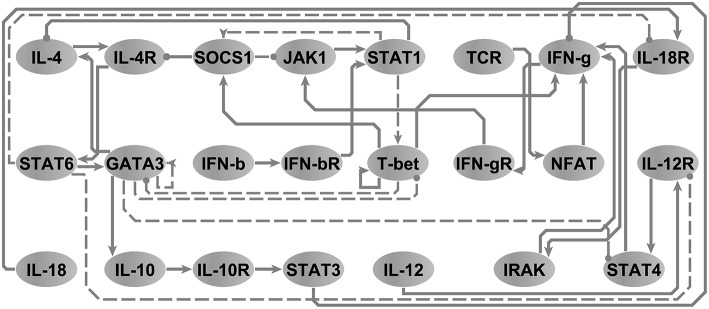
The T-helper network. Arrow-head and circle-head edges indicate activating and inhibiting interactions, respectively. Dashed edges highlight the interactions that were marked by the model checker not necessary in order to reproduce the three steady states reported in Garg et al. (2008). Adapted from Figure 5A, Sedghamiz et al. (2019).

In order to illustrate the utility of our approach in the reduction of models to minimal representations, we analyzed an immune signaling network describing the differentiation of naïve T helper (Th) cells to either a Th1 or Th2 phenotype. This network was composed of 26 molecular and cellular cues (Garg et al., 2008) linked by 35 regulatory interactions (see, Figure S2.2). We asked the model checker what would be the smallest network model in terms of number of interactions capable of reproducing the same 3 attractors supported by the original model without changing any polarity of interactions in the network. Note that node steady states are identical regardless of the state transition update scheme and that accordingly the running times are identical for both scenarios. The model-checker identified a minimal network with 26 interactions (e.g., *z*_1_ = 0.74) and their corresponding logical parameters that exactly reproduced the 3 attractors reported by the original model (see Figure 4). The resultant minimal network model was able to reproduce the documented bi-stability of competition between the master regulatory Th1/Th2 transcription factors T-bet and GATA3 (Fang and Zhu, 2017) in a more parsimonious form. Where the initial model contained direct inhibitory connections in both directions between T-bet and GATA3, we discovered that these direct interactions were not necessary to support the available steady states. Instead, selective activation of T-bet or GATA3 in the reduced model is sustained by positive feedback (direct in the case of T-bet and mediated by IL-4 and STAT6 in the case of GATA3), with activation of either interrupting the other's feedback loop. In order to verify this, we used BoolNet (Mssel et al., 2010) to identify the attractors of the reduced model and obtained identical results. Note that here, the only objective was to minimize the cardinality of the $\hat{P}$ vector, since the network is binary (∀{*i, j*} ∈ *V, w*_*ij*_ ↔ 1) and since the only criteria is to reproduce the node steady states (e.g., (*X*↔*Y*)⇒(*z*_2_ = 0∧*z*_3_ = 0)).

### Recovering Dynamics: The IRMA Gene Network

We also applied the model checker to the synthetic network regulating the expression of 5 genes in yeast known as the IRMA network, a well-studied model in the development of reverse engineering applications (see, Figure S2.3). We asked the model checker to find the most robust model reproducing the knockout measurements provided in Cantone et al. (2009) and translated into discrete data points in Klarner et al. (2012b). The linear optimizers identified a slightly different solution (e.g., though the overall objective function value was lower) than the solution found by the pareto solver. The former solution has a lower robustness but is more efficient (e.g., in terms of the number of transitions needed to reproduce the time series), while the latter is more robust and slightly less efficient suggesting that a Pareto solution might prioritize robustness over efficiency. In terms of run time, *Chuffed* and *OR-tools* showed similar performance with both being at least 7 times faster than the Pareto solver *OptiMathsat*. The parameterization under the synchronous update scheme was unsatisfiable for a bound of up to *M* = 50. *Chuffed* was the fastest solver to prove unsatisfiability (e.g., only ≈ 5 s) followed by *OptiMathSat* illustrating the power of CP solvers in proving unsatisfiability even for large state transition bounds.

### Scalability: The Dendritic Cell Network

In order to show the scalability of our approach to large state spaces, we applied it to a literature-mined network known as Dendritic Cell (Dcell) (Garg et al., 2008) that contains 114 entities connected by 129 interactions (see, Figure S2.4). We generated a synthetic measurement data along a 3-point time course to serve in parameterizing this network. All the techniques listed in Table 2 agreed on a single optimal solution and again in terms of convergence time the sequential *Chuffed* was the fastest. Importantly while this network included the largest number of entities, it was also the most sparsely connected which further exacerbates the network identification problem from experimental data. While regulatory complexity, or the number of regulators at a given node, is a central driver of complexity in parameter identification the overall size of the state transition space is also an important contributor. In this example, regulatory complexity may be moderate however the state space is sizable i.e., |*S*| is of the order of 2^114^. This would be a challenge for exact parameterization methods that explore the complete STG while in this work we show that the bounded parameterization techniques such as the one proposed are able to easily traverse such large STG.

**Table 2:** Computational performance.

**Net**	**Chuffed**							**Google OR-Tools**							**OptiMathSat (Pareto)**						
	**Synch**			**Asynch**				**Synch**			**Asynch**				**Synch**			**Asynch**			
	**T(s)**	**z_**1**_**	**z_**2**_**	**T(s)**	**z_**1**_**	**z_**2**_**	**z_**3**_**	**T(s)**	**z_**1**_**	**z_**2**_**	**T(s)**	**z_**1**_**	**z_**2**_**	**z_**3**_**	**T(s)**	**z_**1**_**	**z_**2**_**	**T(s)**	**z_**1**_**	**z_**2**_**	**z_**3**_**
HPA	0.5	0.61	0	0.6	0.45	0.08	0.11	0.81	0.61	0	0.92	0.61	0.08	0.11	265.3	0.61	0	123.4	0.45	0.08	0.11
IRMA	5.2	†	†	56.1	1	0.2	0.037	715.65	†	†	44.36	1	0.2	0.037	70.23			1800.1	1	0.229	0.035
Dcell	1.8	1	0	2.9	1	0	0.003	31.2	†	†	82.1	1	0	0.003	60.2	1	0	2700.1	1	0	0.003
HPG	3,650	0.46	0.05	^*^	0.45	0.07	0.01	1,020	0.46	0.05	^*^	0.42	0.06	0.01	^*^	^**^	^**^	^*^	0.48	0.10	0.2
Th	0.4	0.74	0	0.4	0.74	0	0	0.5	0.74	0	0.5	0.74	0	0	0.63	0.74	0	0.63	0.74	0	0

*Performance of the proposed method on five biological networks using three solvers, namely Chuffed, OR-tools and OptiMatSat, for synchronous (Sync) and asynchronous (Async) updating schemes. Metrics include the execution time in seconds T(s) as well as the objective function value for structural complexity z_1_, path length z_2_, and path robustness z_3_*.

† *Unsatisfiable, meaning that there existed no parameterization supporting the constraints*.

* *Parameterization tasks that were interrupted because they did not converge within the maximum computation of 9,000 s*.

** *No solution was found within the time limit. For the HPG model none of the models found an optimal solution within the time-limit, reported solutions are the sub-optimal ones obtained within this limit. Note that in some cases a Pareto solution did not exist, in those cases we report the final solution reported by OptiMathSat. Furthermore, the objective values reported in the table are normalized. Bio-ModelChecker normalizes these values by dividing them by the maximum objective achievable in each case*.

### Model Discovery: The Female Hypothalamic-Pituitary-Gonadal (HPG) Axis

Finally, we applied this framework to the recovery and analysis of a minimal representation of female sex hormone regulation by the Hypothalamic-Pituitary-Gonadal (HPG) axis required to reproduce the basic menstrual cycle as previously reported in our earlier work (Sedghamiz et al., 2017). Activation of the HPG axis involves release of Gonadotropin-releasing hormone (GnRH) by the hypothalamus in the mid-brain, prompting the pituitary to then release luteinizing hormone (LH), as well as follicle-stimulating hormone (FSH) into circulation. LH and FSH flow to the gonads, stimulating the ovaries produce estrogen and progesterone which in turn prompt a negative feedback to the hypothalamus down-regulating GnRH release (Viau, 2002). We started with a fully connected network (5 entities, 25 edges) where only 5 interactions were known in terms of polarity and where the characteristics of the remaining interactions were completely unknown (e.g., polarity, threshold of action, and dynamics) (see Figure S2.5). In addition, the time course is cyclic with the last time point returning to the first, making this benchmark network the most challenging model to identify because of its complex dynamic behavior (i.e., limit cycle). Under the asynchronous update scheme none of the solvers found an optimal solution within the time limit but *OR-tools* provided the best solutions in terms of all objectives and the total objective **Z**.

## Discussion

While the significant inroads have been made in the study of biological networks with platforms like CellNOptR (Terfve et al., 2012) and Optimusqual (Dorier et al., 2016) these pioneering tools apply global goal-seeking approaches like genetic algorithms to reconcile experimental data with prior knowledge network dynamics based on Boolean logic and extensions such as constrained fuzzy logic and logic-based ODEs. In this work, we have proposed an extended framework based on multi-level logic where we apply bounded model checking using CSP in order to provide an exhaustive search of the parameter space while addressing the super-exponential nature of this problem in a multi-objective setting, both of which are novel. This work integrates in a modular framework, the latest techniques developed in the Artificial Intelligence (AI) community specifically CSP to the biological model identification problem, making it effortless to employ the latest refinements to these solvers. In an extension of similar tools available for studying logical networks such as TREMPPI, Caspo and GINsim, Bio-ModelChecker directly supports the learning of activation threshold values for regulatory interactions (edge thresholds) from experimental data and also accommodates the use of confidence scores on edges derived from the literature. We show that by choosing smaller bounds for model checking it is possible to reverse engineer relatively large biological networks (e.g., Dcell with 114 entities) in a reasonably efficient computational time. A caveat to this increase in scale remains the degree of regulatory complexity at individual nodes. Indeed, as this method is based on an exhaustive search of all possible combinations of active regulators, the number of discrete values that may be assumed by the logical parameters K increases exponentially with respect to the number of regulators of a given node (e.g., $2^{\left|q_{i}\right|}$, where |***q***_***i***_| is the number of regulators of node *v*_*i*_). Therefore, regardless of the parameterization technique employed, due to the formalism definition itself, the number of regulators may be expected to be a limiting factor, especially as it increases beyond 15. However, as our proposed parameterization technique is based on constraint satisfaction programming (CSP), we submit that it is especially well-suited to such complexity and may nonetheless continue to support complete searches within reasonable execution times even in networks populated by nodes with 5 or more concurrent regulators.

In addition to offering an attractive algorithmic compromise between completeness of search and network complexity, we introduced here in the formulation of the problem itself several biologically inspired measures of optimality such as efficiency of regulatory structure, robustness of response, and path length cost that may serve in ranking families of feasible models according to their plausibility. For instance, the reduction of models to their simplest representation by focusing on the minimization of the first objective alone translates into identifying the set of most crucial interactions in a network that are necessary to reproduce a set of steady states or temporal behavior. With regard to better understanding the design principles of biological signaling, a multi-objective view of these problems opens new doors for further research. Specifically, applying these objective functions separately to well-studied biological systems would allow us to further explore how signal transduction efficiency is balanced against signal robustness and how this trade-off may be weighted differently according to biological function and level of biology. In this work we define increased robustness as a reduction in the branching of dynamic response offered by competing state transitions. Though some level of kinetic stochasticity remains a legitimate feature at certain levels of biology, we present the limit case where no information regarding the relative kinetics of any state variable is available and the system evolves according to a completely asynchronous update. This is an extreme case that almost invariably supports trajectories that are biologically infeasible. Indeed, in previous work (Sedghamiz et al., 2018) we have shown that grouping state variables according to physiological compartment and assigning a priority of update based on the relative kinetics of these groups greatly reduces the complexity of the state transition graph and significantly improves biological fidelity. Nonetheless as stochasticity remains a basic feature of biology, we submit that this measure of signal robustness will remain of interest even in well-characterized systems and that the identification of regulatory characteristics that accommodate such stochasticity while also imparting an increasing consistency in behavior will offer new insights into these systems. Importantly, this framework is the only method that to our knowledge offers this type of parameterization in two different updating schemes namely synchronous and asynchronous. The parameterization of the models under synchronous update being more computationally efficient, it is useful to employ this update scheme for initial exploration of much larger models. Moreover, being able to reverse engineer the biological models based on two different state transition update schemes might allow a better understanding of dependency of the network on time delays associated with the entities involved.

## Author Contributions

HS developed and evaluated the mathematical analysis tools, ran simulations, prepared graphics, and drafted the initial manuscript. MM helped design and select the biological models, and contributed to the biological interpretations in the manuscript. TC contributed to prior work with the model and reviewed the manuscript. DW guided core algorithmic changes leading to significant increases in efficiency and reviewed the manuscript. GB directed the work, contributed directly to the development of the original, and revised frameworks, and was a major contributor in writing the manuscript. All authors read and approved the final manuscript.

### Conflict of Interest Statement

The authors declare that the research was conducted in the absence of any commercial or financial relationships that could be construed as a potential conflict of interest.

## Supplementary Material

BioModelChecker (BioMC), a standalone software developed by our group for the reverse engineering and analysis of regulatory networks based on Constraint Satisfaction (CS) techniques is freely available at https://github.com/hooman650/BioModelChecker. Also available at this site are accompanying user documentation and example problems. Supplementary Material includes the details regarding the derivation of propositional formulas in Equations (2) and (3).

Supplementary Material File 1 Detailed derivation of update schemes.

Supplementary Material File 2 Model regulatory circuits; Figures S2.1–5.
