# Supplementary material for: Bio-ModelChecker: Using Bounded Constraint Satisfaction to Seamlessly Integrate Observed Behavior With Prior Knowledge of Biological Networks
Source: Front Bioeng Biotechnol. 2019 Mar 26;7:48. doi: 10.3389/fbioe.2019.00048 (PMC6443719; doi:10.3389/fbioe.2019.00048)
Supplement: Supplementary Material File 1 — Detailed derivation of update schemes. [file Data_Sheet_1.pdf]

Supplementary Material:

On Parametrization of Multi-valued Regulatory Networks

Hooman Sedghamiz, Matthew Morris, Travis JA Craddock, Darrell Whitley and Gordon Broderick*

*Correspondence:
Gordon Broderick, PhD
gordon.broderick@rochesterregional.org

1 PROPOSITIONAL FORMULA FOR TIME UPDATES.

Lemma 1. Let $\mathbf{x^t} = \{x_1^t, x_2^t, \dots, x_n^t\}$ be a vector representing the state of a regulatory network, TR_{MxN} be a trajectory consisting M states and N entities where $\mathbf{x^t}$ is the state of the network at time t. The logical equation governing such a trajectory depends on the choice of update scheme is may be defined for the classical synchronous and asynchronous update schemes as follows:

1. Under synchronous update:

$$TR_{1\dots M}^{Synch} = \wedge_{t=1}^{M-1} \wedge_{i=1}^{N} T_i^{Synch}(\boldsymbol{x^t}, \boldsymbol{x^{t+1}}) \quad \text{(S1)}$$

2. Under asynchronous update:

$$TR_{1\dots M}^{Asynch} = \wedge_{t=1}^{M-1} \vee_{i=1}^{N} T_i^{Asynch}(\boldsymbol{x^t}, \boldsymbol{x^{t+1}}) \quad \text{(S2)}$$

Where $\boldsymbol{T_i^{Synch}}$ and $\boldsymbol{T_i^{Asynch}}$ represent the transition for node v_i (where $i \in \{1, N\}$) given its image vector $\boldsymbol{Y}$ and current state of the network $\boldsymbol{x^t}$ under synchronous and asynchronous update respectively.

Proof. 1.1: Under the synchronous update assumption, two criteria should be satisfied:

1. First, entities are only able to change their expression levels in a step-wise manner. Therefore, a variable SC_i is defined to denote the gradient of state change for node v_i from its current state x_i towards its image y_i as:

$$SC_i = \begin{cases} 1 & if\ y_i > x_i^t \\ -1 & if\ y_i < x_i^t \\ 0 & if\ y_i = x_i^t \end{cases} \quad \text{(S3)}$$

Then the state transition T_i^{Synch} for node v_i from its current state x_i^t to its next state x_i^{t+1} may be expressed as:

$$T_i^{Synch}(\mathbf{x^t}, \mathbf{x^{t+1}}) = (x_i^t \leftrightarrow (x_i^{t+1} + SC_i)) \tag{S4}$$

2. All the nodes are able to change their expression level simultaneously which can be denoted by taking the conjunction between all of the state variables;

$$\mathbf{T^{Synch}}(\mathbf{x^t}, \mathbf{x^{t+1}}) = \wedge_{i=1}^{N} T_i^{Synch}(\mathbf{x^t}, \mathbf{x^{t+1}}) \tag{S5}$$

Consequently, the state of the network after *M* iterations forms a trajectory containing a conjunction of *M-1* states as;

$$\begin{aligned}\mathbf{T^{Synch}_{1...M}} &= \left(\bigwedge_{i=1}^{N} T_i^{Synch}(\mathbf{x^1}, \mathbf{x^2})\right) \wedge \left(\bigwedge_{i=1}^{N} T_i^{Synch}(\mathbf{x^2}, \mathbf{x^3})\right) \wedge \ldots \left(\bigwedge_{i=1}^{N} T_i^{Synch}(\mathbf{x^{M-1}}, \mathbf{x^M})\right) \\ &= \bigwedge_{t=1}^{M-1} \bigwedge_{i=1}^{N} T_i^{Synch}(\mathbf{x^t}, \mathbf{x^{t+1}})\end{aligned} \tag{S6}$$

Proof. 1.2: Under an asynchronous update assumption, four criteria should be satisfied:

1. A step change SC_i is defined for node v_i similar to the synchronous update in Eq. S3.

2. However, under asynchronous update, only one node v_i can exercise a transition to its next image state y_i at a given time step such that the *partial* update TP_i^{Asynch} becomes:

$$TP_i^{Asynch}(\mathbf{x^t}, \mathbf{x^{t+1}}) = (x_i^{t+1} \leftrightarrow (x_i^t + SC_i) \wedge \wedge_{j \neq i}(x_j^{t+1} \leftrightarrow x_j^t) \tag{S7}$$

3. A state can undergo a self-transition *iff* none of the variables are called upon to change state. First a binary variable is defined to check whether any of the variables have an opportunity to change:

$$F(\mathbf{x^t}) = \wedge_{i=1}^{N}(y_i \odot x_i^t) \tag{S8}$$

where ⊙ is the multivalued XNOR operator (e.g. 2 ⊙ 2 = true). $F(x^t)$ is true if the state of all variables from the time step *t* and their corresponding image state are equal. Then the general case of a transition for variable v_i is defined as,

$$T_i^{Asynch}(\mathbf{x^t}, \mathbf{x^{t+1}}) = \{F(\mathbf{x^t}) \vee (y_i \oplus x_i^t)\} \wedge TP_i^{Asynch}(\mathbf{x^t}, \mathbf{x^{t+1}}) \tag{S9}$$

where ⊕ is the multivalued XOR operator (e.g. 2 ⊕ 1 = true; 3⊕ 3 = false). Eq. S9 states that a transition is possible when either none of the nodes change their expression level (e.g. $F(\mathbf{x^t})$ = *true*) or when only node v_i is eligible to change its state ($y_i \oplus x_i^t = true$).

4. Finally, when each variable is equally likely to change its expression level the overall state transition is represented as a disjunction between all of the variables in the network:

$$\mathbf{T}^{\mathbf{Asynch}}(\mathbf{x}^{\mathbf{t}}, \mathbf{x}^{\mathbf{t+1}}) = \bigvee_{i=1}^{N} T_i^{Asynch}(\mathbf{x}^{\mathbf{t}}, \mathbf{x}^{\mathbf{t+1}}) \quad \text{(S10)}$$

Similar to the synchronous case (see Eq. S6), the state of the network after *M* iterations forms a trajectory containing conjunction of *M*-1 states which results in Eq. S2.
