# Supplementary material for: Bio-ModelChecker: Using Bounded Constraint Satisfaction to Seamlessly Integrate Observed Behavior With Prior Knowledge of Biological Networks
Source: Front Bioeng Biotechnol. 2019 Mar 26;7:48. doi: 10.3389/fbioe.2019.00048 (PMC6443719; doi:10.3389/fbioe.2019.00048)
Supplement: Supplementary Material File 2 — Model regulatory circuits; Figures S2.1–5. [file Data_Sheet_2.PDF]

Supplementary Material:

On Parametrization of Multi-valued Regulatory Networks

Hooman Sedghamiz, Matthew Morris, Travis JA Craddock, Darrell Whitley and Gordon Broderick*

*Correspondence:
Gordon Broderick, PhD
gordon.broderick@rochesterregional.org

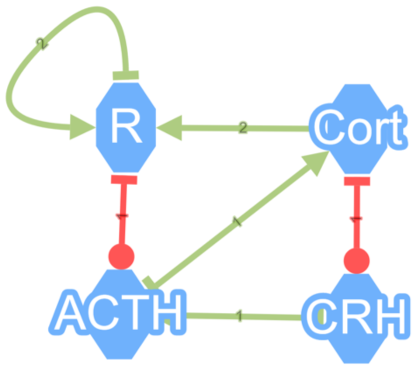

Figure S2.1. Regulatory circuit diagram for the Hypothalamic-Pituitary-Adrenal axis (Section 4.1).

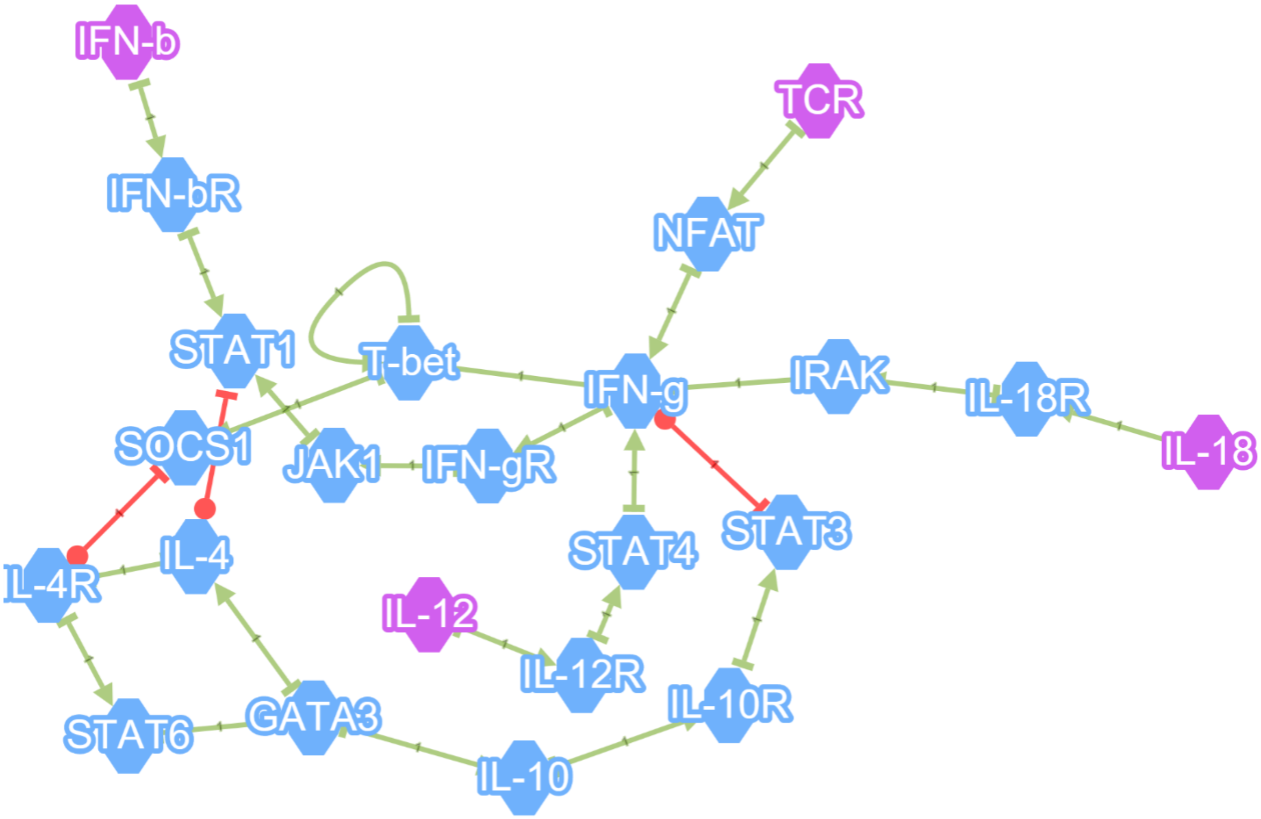

Figure S2.2. Regulatory circuit diagram for the T-helper cell model (Section 4.2).

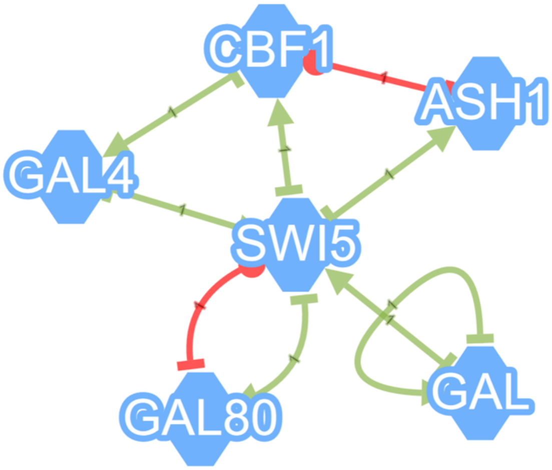

Figure S2.3. Regulatory circuit diagram for the IRMA model (Section 4.3).

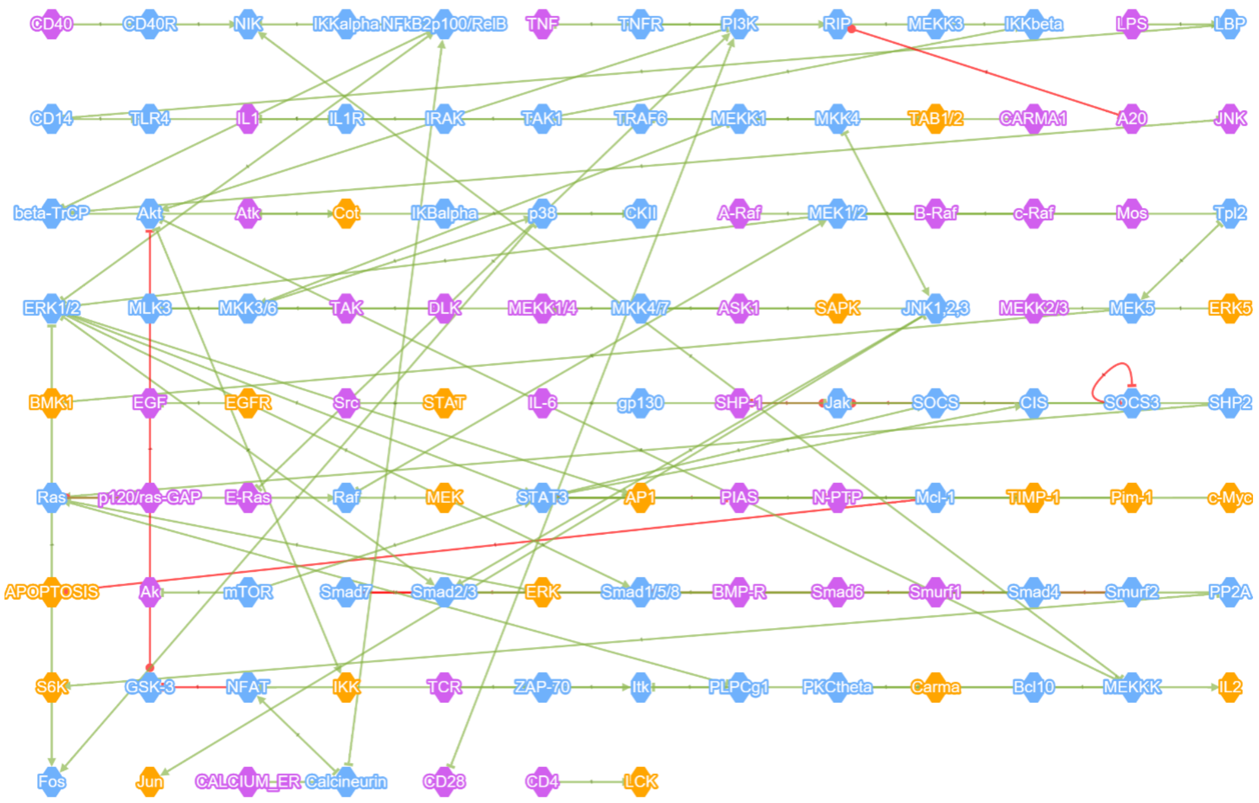

Figure S2.4. Regulatory circuit diagram for the dendritic cell (DCell) model (Section 4.4).

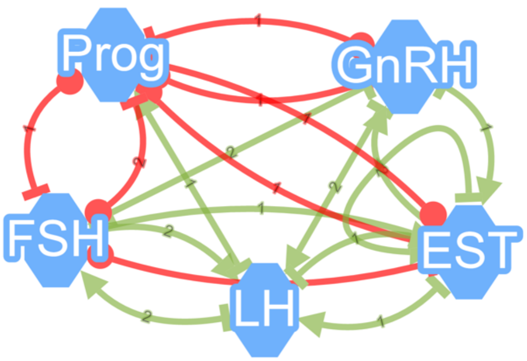

Figure S2.5. Regulatory circuit diagram for control of female sex hormones via the Hypothalamic-Pituitary-Gonadal (HPG) axis (Section 4.5).
